# P12 Focal myositis—A case series of a rare cause of hip immobility and calf pseudotumor in children

**DOI:** 10.1093/rap/rkab068.011

**Published:** 2021-10-19

**Authors:** Fahim Patel, Arani Sridhar

**Affiliations:** Department of Paediatric Rheumatology, University Hospitals of Leicester, Leicester, United Kingdom

## Abstract

**Case report - Introduction:**

Focal myositis is a rare immune-mediated pseudotumour of a single skeletal muscle group^.^ Only around 200 cases have been described in the literature, so little is known about incidence, prevalence, patient management and outcomes. This differs and should not be confused with post-viral myalgia which bears neither the histological changes nor chronicity of focal myositis. Treatment options are centred on immunomodulation and in severe cases surgical management of contractures.

**Case report - Case description:**

Case 1

A systemically well 7-year-old girl presented with 5-weeks of right calf tenderness and swelling following a short episode of pharyngitis and generalised maculopapular rash. There was no gait abnormality, focal neurology or restriction in activity aside from fatigability on walking distances. There were no skin rashes, joint involvement, eye changes or involvement of other muscles. She had a raised creatine kinase, plasma viscosity and lactate dehydrogenase. Her other blood results were normal including an extended autoimmune screen, immunoglobulins, complement levels, ASOT and titres of mycoplasma, EBV and CMV. MRI showed evidence of extensive inflammation of the gastrocnemius and soleus. A muscle biopsy showed heavy interstitial inflammatory cell infiltrate of predominantly lymphocytes, features of fibre necrosis including phagocytosis and hyalinisation with concurrent fibre regeneration.

Case 2

A systemically well 14-year-old presented with 6-months of left-sided hip pain, weight loss and inability to weight-bear without crutches. On examination there was painful fixed limitation of the left hip to 45^o ^on abduction and external rotation with bilateral mild swelling of the proximal interphalangeal (PIP) joints on both upper limbs. Otherwise, there was a full range of movement in all joints, with no rashes or other joint swelling or inflammation. Her blood tests were ANA positive 1:6000 and MRI of her hips demonstrated high T2 signal intensity in the left gluteus minimis and medius, obturator internus, obturator externus in keeping with myositis.

**Case report - Discussion:**

Case 1

She was initially managed with physiotherapy and anti-inflammatory medications but then developed intermittent right calf pain, restriction in activity and tiptoe walking due to gastrocnemius contractures. She was commenced on an 8-week tapering course of oral steroids and is improving with weekly methotrexate.

Case 2

She received a pulse of corticosteroids followed by a course of methotrexate. There was immediate improvement in her PIP joint swellings and within a few weeks she was able to walk without crutches for the first time in 6 months; a surveillance MRI confirmed complete radiographical resolution of myositis. Unfortunately, 18 months after her diagnosis, she had developed anterior uveitis of her left eye with posterior synechiae; this responded well to steroid and cyclopentolate eye drops.

**Case report - Key learning points:**

We emphasise that clinicians should bear this rare differential diagnosis for in mind for consideration of early conservative management, assessment for uveitis, immunomodulation and possibly surgical correction to improve patient outcome.

